# Unique Patterns and Biogeochemical Relevance of Two-Component Sensing in Marine Bacteria

**DOI:** 10.1128/mSystems.00317-18

**Published:** 2019-02-05

**Authors:** Noelle A. Held, Matthew R. McIlvin, Dawn M. Moran, Michael T. Laub, Mak A. Saito

**Affiliations:** a MIT-WHOI Joint Program in Oceanography/Applied Ocean Science and Engineering, Woods Hole, Massachusetts, USA; b Marine Chemistry and Geochemistry Department, Woods Hole Oceanographic Institution, Woods Hole, Massachusetts, USA; c Department of Biology, Massachusetts Institute of Technology, Cambridge, Massachusetts, USA; d Howard Hughes Medical Institute, Massachusetts Institute of Technology, Cambridge, Massachusetts, USA; Florida State University

**Keywords:** biogeochemistry, cell signaling, gene regulation, marine microbiology, proteomics, regulatory network, two-component system

## Abstract

Two-component sensory (TCS) systems link microbial physiology to the environment and thus may play key roles in biogeochemical cycles. In this study, we surveyed the TCS systems of 328 diverse marine bacterial species. We identified lifestyle traits such as copiotrophy and diazotrophy that are associated with larger numbers of TCS system genes within the genome. We compared marine bacterial species with 1,152 reference bacterial species from a variety of habitats and found evidence of extra response regulators in marine genomes. Examining the location of TCS genes along the circular bacterial genome, we also found that marine bacteria have a large number of “orphan” genes, as well as many hybrid histidine kinases. The prevalence of “extra” response regulators, orphan genes, and hybrid TCS systems suggests that marine bacteria break with traditional understanding of how TCS systems operate. These trends suggest prevalent regulatory networking, which may allow coordinated physiological responses to multiple environmental signals and may represent a specific adaptation to the marine environment. We examine phylogenetic and lifestyle traits that influence the number and structure of two-component systems in the genome, finding, for example, that a lack of two-component systems is a hallmark of oligotrophy. Finally, in an effort to demonstrate the importance of TCS systems to marine biogeochemistry, we examined the distribution of *Prochlorococcus/Synechococcus* response regulator PMT9312_0717 in metaproteomes of the tropical South Pacific. We found that this protein’s abundance is related to phosphate concentrations, consistent with a putative role in phosphate regulation.

**IMPORTANCE** Marine microbes must manage variation in their chemical, physical, and biological surroundings. Because they directly link bacterial physiology to environmental changes, TCS systems are crucial to the bacterial cell. This study surveyed TCS systems in a large number of marine bacteria and identified key phylogenetic and lifestyle patterns in environmental sensing. We found evidence that, in comparison with bacteria as a whole, marine organisms have irregular TCS system constructs which might represent an adaptation specific to the marine environment. Additionally, we demonstrate the biogeochemical relevance of TCS systems by correlating the presence of the PMT9312_0717 response regulator protein to phosphate concentrations in the South Pacific. We highlight that despite their potential ecological and biogeochemical relevance, TCS systems have been understudied in the marine ecosystem. This report expands our understanding of the breadth of bacterial TCS systems and how marine bacteria have adapted to survive in their unique environment.

## Abstract (precis)

Marine microbes must manage variation in their chemical, physical, and biological surroundings. Because they directly link bacterial physiology to environmental changes, TCS systems are crucial to the bacterial cell. This study surveyed TCS systems in a large number of marine bacteria and identified key phylogenetic and lifestyle patterns in environmental sensing. We found evidence that, in comparison with bacteria as a whole, marine organisms have irregular TCS system constructs which might represent an adaptation specific to the marine environment. Additionally, we demonstrate the biogeochemical relevance of TCS systems by correlating the presence of the PMT9312_0717 response regulator protein to phosphate concentrations in the South Pacific. We highlight that despite their potential ecological and biogeochemical relevance, TCS systems have been understudied in the marine ecosystem. This report expands our understanding of the breadth of bacterial TCS systems and how marine bacteria have adapted to survive in their unique environment.

## INTRODUCTION

A bacterium’s survival is dependent on its ability to respond to changes in its environment. This is especially true for marine microbes, which experience changes in nutrient availability, light, temperature, and community structure that can occur on time scales as short as hours (1). Two-component sensory (TCS) systems, which modulate gene expression on short time scales, are the most common sensory systems in prokaryotes (2). The organism’s complement of two-component-system genes may thus reveal details about its lifestyle, ecological niche, and physiological complexity.

**FIG 1:**
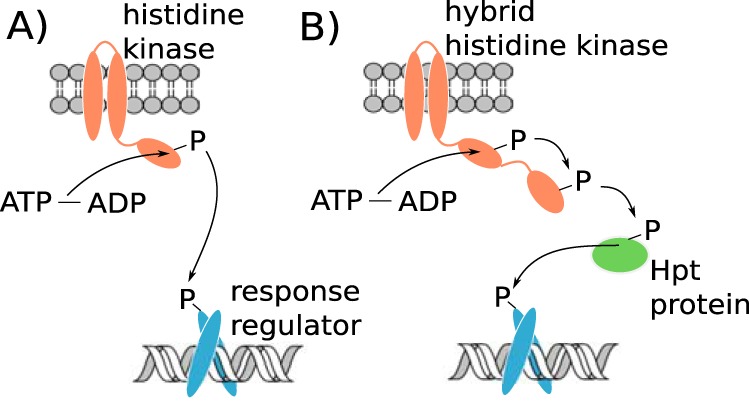
Overview of two-component-system signaling in (A) a traditional histidine kinase-response regulator system and (B) a hybrid histidine kinase system. In panel A, the phosphorylation is transferred from the histidine kinase to the response regulator by a direct protein-protein interaction. In panel B, the phosphorylation is transferred to an internal receiver domain on the histidine kinase, then to one or more histidine phosphotransfer (Hpt) proteins, and finally to the terminal response regulator.

Canonically, two-component sensory systems are composed of a single histidine kinase and response regulator protein pair (Fig. 1). The histidine kinase contains a sensory domain that is activated by a specific stimulus, which may be a small molecule, nutrient, or physical property such as light or temperature. Upon activation, the histidine kinase is autophosphorylated on a conserved histidine residue, inducing conformational changes that enhance interactions with the response regulator protein (3). Through a highly specific protein-protein interaction, the phosphate moiety is transferred from the histidine kinase to a conserved aspartate residue on the response regulator (4). This typically stimulates binding of the response regulator to a DNA promoter region, resulting in transcription of genes in the downstream operon (5). A variation on this model is the hybrid histidine kinase, in which the kinase and response regulator are located on a single protein. An intermediary protein or set of proteins is involved in transmitting the signal, allowing multiple levels of control and a fine-tuned physiological response (3).

Two-component sensory systems represent a direct link between the environment and the physiology of the prokaryotic cell and, as such, may be important players in biogeochemical cycles. One well-studied example is the Pho system, composed of the histidine kinase PhoR and response regulator PhoB. The Pho system is common in marine bacteria and regulates genes that are involved in phosphate acquisition (6). In *Prochlorococcus*, activation of PhoR by low intracellular phosphate stimulates transcription of alkaline phosphatase. Alkaline phosphatase cleaves phosphate from organic matter, providing a source of phosphate that would otherwise be inaccessible (7). As the vector linking microbial physiology (i.e., the expression of alkaline phosphatase enzyme) to ocean chemistry (i.e., phosphate availability), the Pho system may be a key regulator of phosphorus cycling in the ocean. Other two-component sensory (TCS) systems may also play important roles in mediating microbe-environment interactions, though we do not yet understand their biogeochemical contexts.

In this study, we surveyed the TCS system genes of 328 diverse marine bacteria, identifying phylogenetic and lifestyle factors that correlate with greater numbers of sensory genes. We compared these marine bacteria to a curated reference collection of 1,152 bacterial genomes, most derived from the GEBA initiative (8). This allowed us to identify key differences in how TCS systems are structured in marine bacteria versus bacteria in general. To demonstrate the importance of TCS systems to marine biogeochemistry, we examined the distribution of a putative phosphate-sensing *Prochlorococcus* response regulator (PMT9312_0717) in metaproteomes from the tropical Pacific. We highlight gaps in our knowledge of marine TCS and emphasize the importance of TCS to our overall understanding of marine bacteria.

## RESULTS

### Lifestyle influences on TCS gene abundance.

We examined the genomes of 328 diverse marine bacteria publicly available in the JGI IMG data warehouse (see Table S1 in the supplemental material). The data set emphasizes cultivable and oceanographically important organisms such as *Prochlorococcus*, *Synechococcus*, *Pelagibacter*, *Alteromonas*, and *Roseobacter*. In total, 15 phyla and 183 genera are represented from a variety of habitats, including coastal ecosystems, the open ocean, hydrothermal vent systems, host-associated environments, and marine sediments. All genomes were labeled high-quality finished or permanent drafts. TCS system genes were identified using highly conserved protein family domains for histidine kinases or response regulators as described in Materials and Methods.

10.1128/mSystems.00317-18.3 TABLE S1 TCS gene data for the 328 marine bacteria surveyed, including taxonomic information for each genome. Download Table S1, XLSX file, 0.2 MB. Copyright © 2019 Held et al. 2019 Held et al. This content is distributed under the terms of the Creative Commons Attribution 4.0 International license.

**FIG 2:**
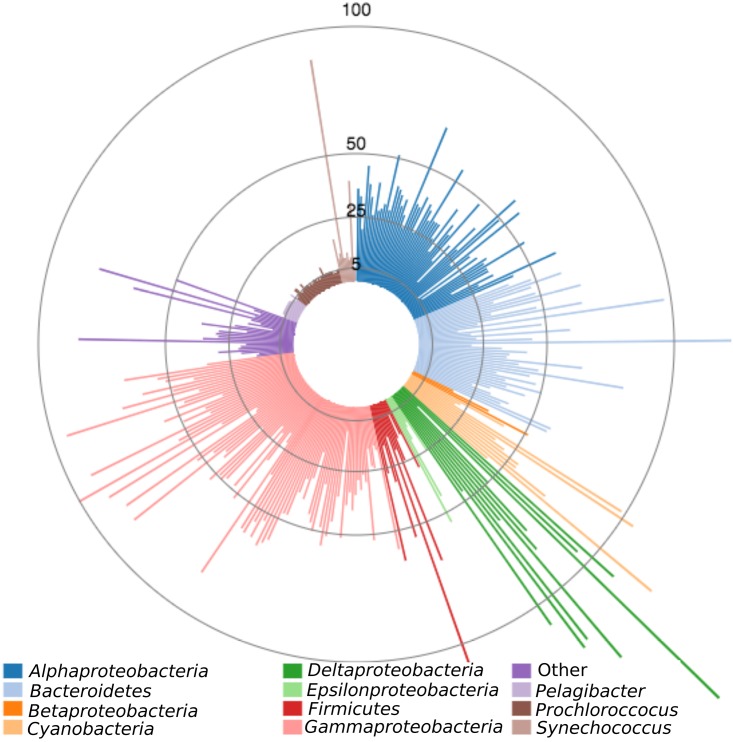
Number of histidine kinase sensory genes in the genomes of 328 diverse marine bacterial species (scale indicated by concentric circles). Phylogenetic groups of interest are delineated by color. The number of histidine kinases in the data set ranges from 1 (*Pelagibacter*) to 174 (Desulfovibrio inopinatus DSM 10711).

We began by examining one half of the two-component system—the sensory histidine protein kinase (HPK). The number of histidine kinases in each genome ranges from 1 (*Pelagibacter*) to 174 (Desulfovibrio inopinatus DSM 10711) and is related to genome size; we found on average 0.902 histidine kinases (HPK) per 100 protein-encoding genes (Fig. 2). A K-means clustering analysis was performed on the proportion of the genome devoted to histidine kinases (expressed for human readability as the number of histidine kinases per 100 protein-encoding genes) versus genome size. This analysis revealed that the number of histidine kinases per 100 protein-encoding genes is dependent on both phylogeny and lifestyle. The first cluster contains oligotrophic picoplankton such as *Prochlorococcus*, *Pelagibacter*, and open ocean *Synechococcus*. These organisms have very small genomes and few histidine kinases per 100 protein-encoding genes. Cluster 2 contains *Rhodobacter*, *Vibrio*, coastal *Synechococcus*, and *Alteromonas* and *Pseudoalteromonas* genomes, which are larger and have more histidine kinases per 100 protein-encoding genes. Cluster 3 is composed mainly of *Alteromonas* and *Pseudoalteromonas* genomes that have genome sizes similar to those of cluster 2 organisms but more histidine kinases per 100 protein-encoding genes. Cluster 4 organisms have the greatest numbers of histidine kinases per 100 protein-encoding genes; many have specific lifestyle traits such as particle association, parasitism, and mat formation. A number are sulfate-reducing *Deltaproteobacteria*.

**FIG 3:**
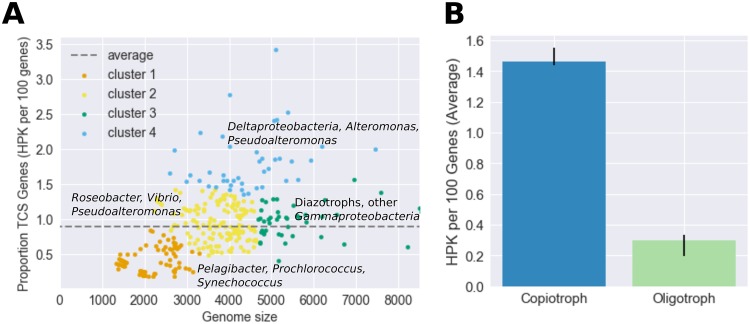
(A) K-means clustering of the number of histidine kinases (HPK) per 100 protein-encoding genes as a function of genome size. The dashed line represents the average, 0.902 histidine kinases per 100 protein-encoding genes in the genome. (B) The number of histidine kinases per 100 protein-encoding genes in the genome for organisms unambiguously designated copiotrophs or oligotrophs. Error bars represent 95% confidence intervals of the average value within the copiotroph (*n* = 74) and oligotroph (*n* = 34) categories. The copiotrophs were shown to have significantly more histidine kinases per gene than the oligotrophs by a Student's *t* test (*P* = 3e^−15^).

Marine organisms are often classified by their nutritional preferences as copiotrophs (adapted to high-nutrient conditions), oligotrophs (organisms adapted to low-nutrient conditions), or as organisms that fall between those classifications (9–12). This provides a framework for understanding properties such as growth rate, cell size, and genome size (Table 1). We classified marine bacteria as copiotrophs or oligotrophs based on published isolation and laboratory growth conditions and found that the copiotrophs have significantly more histidine kinases per gene than the oligotrophs (*P* = 3e^−15^ by Student's *t* test) (Fig. 3B).

**TABLE 1:** Characteristics of copiotrophs versus oligotrophs and their TCS system genes^*a*^

Genus	Example genome size (bp)	Example % growth rate (per day)	Lifestyle(s)	HPK/100 genes	RR/HPK ratio	% hybrid HPKs	Reference
*Pelagibacter*	1,200–1,400	0.4–0.58	Oligotroph	0.387	0.83	Typically 0	59
*Prochlorococcus*	1,200–2,000	0.51–0.83	Oligotroph	0.76	1.22	Typically 0	60
*Synechococcus*	1,500–3,000	1	Oligotroph	1.005	1.23	0–40	60
*Trichodesmium*	∼5,000	0.29	Oligotroph	0.694	0.753	15–35	61
*Crocosphaera*	∼6,000	0.5	Oligotroph	0.723	0.992	∼35	62
*Roseobacter*	∼5,000	1.45	Varies/copiotroph	0.755	0.991	10–40	63
*Vibrio*	∼5,000	Up to 14.3	Copiotroph	1.25	1.07	25–50	64
*Alteromonas*	4,000–4,500	6	Copiotroph	1.43	1.06	∼40	65
*Pseudoalteromonas*	3,000–5,000	∼30	Copiotroph	1.5	1.1	40–50	66

a HPK, histidine kinase; RR, response regulator.

### Unusual patterns in marine TCS sensing genes.

We compared the TCS system genes of marine bacteria with those of 1,152 reference bacteria (Table S2). The reference data set includes bacteria from diverse lineages and habitats, including terrestrial, freshwater, host-associated, and marine bacteria, and is representative of trends in the two-component systems of bacteria as a whole. The phylogenetic distribution of genomes in the reference data set is broad (see Fig. S1 in the supplemental material). An important caveat is that both the marine and reference data sets contained mainly cultured organisms and may not represent natural diversity (13).

10.1128/mSystems.00317-18.1 FIG S1 Phylogenetic breakdown of the marine and reference data sets, showing the diversity of the genomes used in this analysis. Download FIG S1, EPS file, 0.09 MB. Copyright © 2019 Held et al. 2019 Held et al. This content is distributed under the terms of the Creative Commons Attribution 4.0 International license.

10.1128/mSystems.00317-18.4 TABLE S2 TCS gene data for the 1,152 reference bacteria, including taxonomic information for each genome. Download Table S2, XLSX file, 0.2 MB. Copyright © 2019 Held et al. 2019 Held et al. This content is distributed under the terms of the Creative Commons Attribution 4.0 International license.

**FIG 4:**
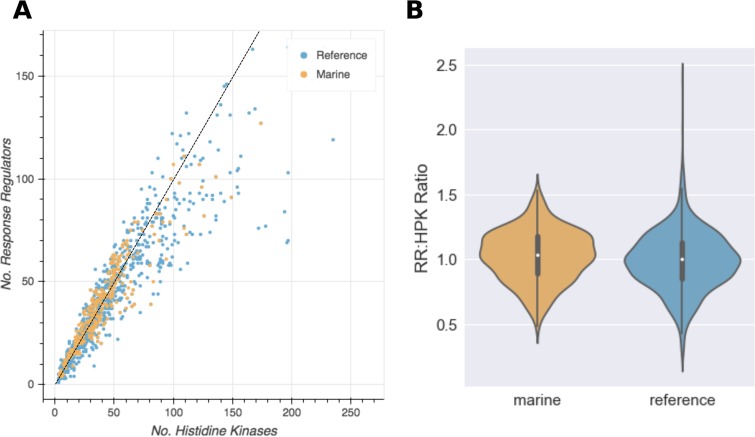
(A) Number of response regulator (RR) genes versus histidine kinase (HPK) genes in marine (orange) and reference (blue) bacteria. The dotted black line represents a 1:1 relationship. When the number of TCS systems is low, the ratio of RRs to HPKs is approximately 1. When the number of two-component systems is large (50 or more), the RR-to-HPK ratio tends to be much lower than 1. (B) RR/HPK ratio of marine and reference bacteria. While the differences are subtle, the marine bacteria have a significantly larger RR/HPK ratio on average (1.03) than the reference bacteria (0.99) as shown by a one-tailed ANOVA test [*P* = 0.0095, F(327,1151) = 6.73].

Based on the traditional understanding, the numbers of histidine kinase and response regulator genes are expected to be equal. Indeed, we found the average response regulator/histidine kinase (RR/HPK) ratio in the reference data set to be 0.99 (Fig. 4). The RR/HPK ratio was found to be slightly higher in the marine bacteria (1.03), suggesting that there are a small number of “extra” response regulators in the genomes. The difference between the marine and reference data sets is significant based on a one-way analysis of variance (ANOVA) test [*P* = 0.009, F(372, 1151) = 6.73].

**FIG 5:**
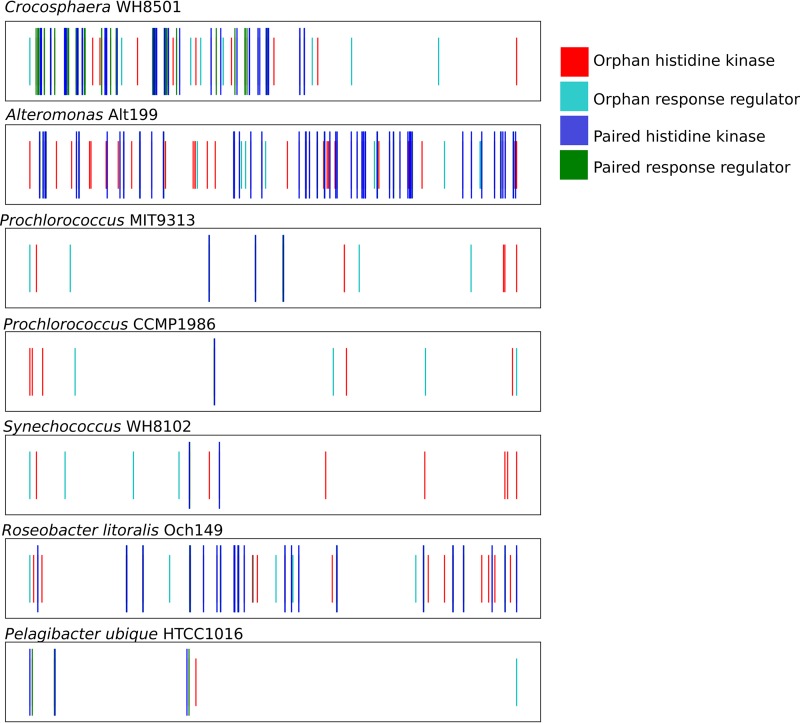
Distribution of TCS genes in various marine bacterial species. The genome is linearized and depicted as a number line; genes are represented as vertical lines based on their starting location. Histidine kinase genes and response regulator genes that are within four genes of another TCS gene are represented as long blue and green lines, respectively. Orphan histidine kinases and response regulators are represented as short cyan and red lines, respectively. There are many orphan TCS genes in marine bacteria, including in oligotrophs such as *Pelagibacter* and *Prochlorococcus*.

To better understand the origin of the high RR/HPK ratio, we examined the locations of the TCS genes in a subselection of marine genomes (Fig. 5). Genomes were selected for their oceanographic relevance, quality, and representation in the literature. Typically, the TCS system histidine kinase and response regulator genes are located in the same operon (14). “Orphan” genes are defined here as genes that are more than four average gene lengths away from another TCS gene. These genes may participate in regulatory networks with other TCS systems (15).

Orphan TCS genes were identified in all seven of the genomes that we examined, consistent with the high RR/HPK ratios described above. For example, the Pelagibacter ubique HTCC1016 genome has three modular TCS systems plus one orphan HPK (a KipI family gene) and one orphan RR (RegB). Notably, KipI is known to participate in regulatory networks (15). Larger genomes have more orphan genes. For instance, *Alteromonas* sp. Alt199 has 26 orphan histidine kinases (30% of the histidine kinase genes) and 8 orphan response regulators (14% of the response regulator genes). *Crocosphaera* sp. WH8501 has 8 orphan histidine kinases (15%) and 9 orphan response regulators (17%). It is difficult to ascertain the function of orphan genes due to the lack of experimental evidence associated with them. These systems are ideal for future study.

**FIG 6:**
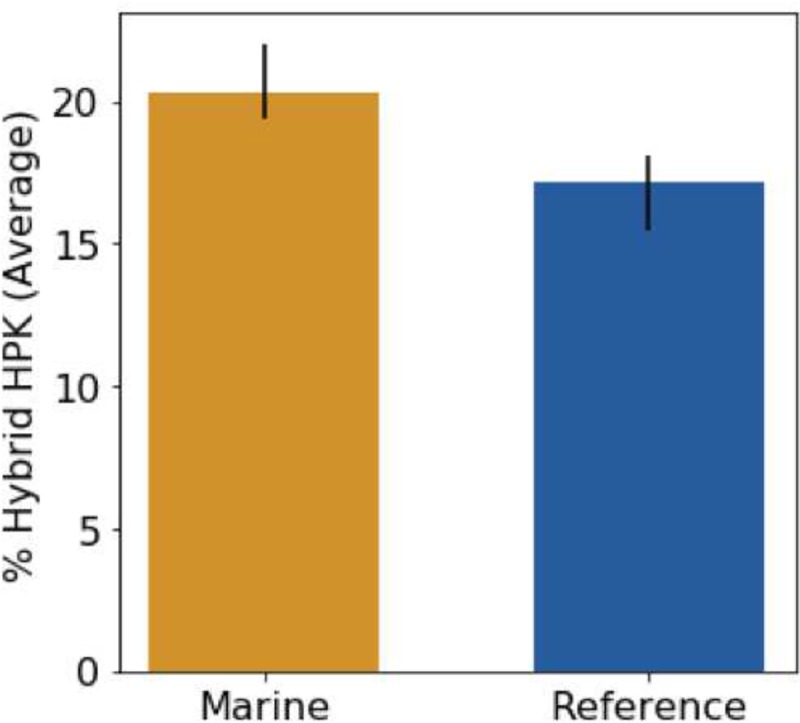
Percentage of histidine kinases that are hybrids in marine (orange) and reference (blue) bacteria. The marine bacteria have a greater percentage of hybrid histidine kinases than the reference bacteria. Error bars represent a bootstrapped 95% confidence interval. The difference is statistically significant by a Student's *t* test (*P* = 3e^−10^).

Hybrid systems occur when the histidine kinase and the response regulator are located on a single protein; here they were identified as genes containing both a histidine kinase HAMP domain and a response regulator receiver domain. In marine bacteria, the proportion of hybrid histidine kinases relative to total histidine kinase content ranged from zero to 61% (Fig. 6). Marine bacteria have significantly more hybrid histidine kinases than reference bacteria (*P* = 3e^−10^ by a Student's *t* test).

### Patterns in *Proteobacteria* and *Cyanobacteria*.

**FIG 7:**
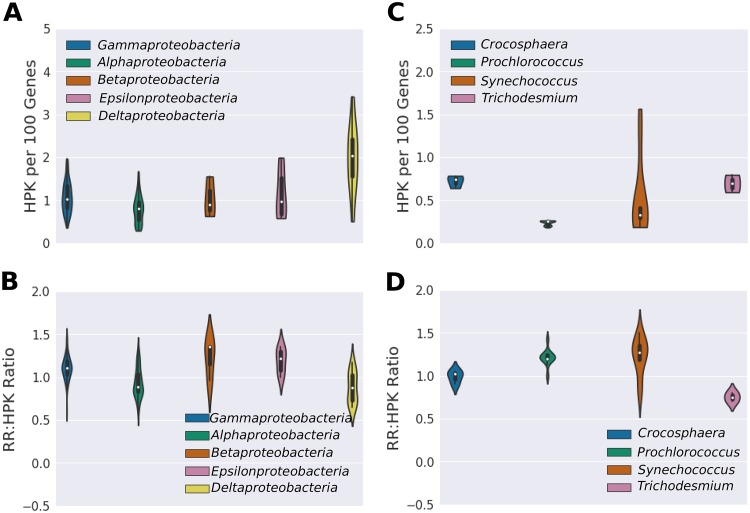
(A) Number of histidine kinases per 100 protein-encoding genes and (B) RR/HPK ratio of *Proteobacteria*. (C) Number of histidine kinases per 100 protein-encoding genes and (D) RR/HPK ratio of *Cyanobacteria*. *Proteobacteria*, in particular the *Deltaproteobacteria*, have many histidine kinases per gene compared with the *Cyanobacteria*. The RR/HPK ratio of the *Proteobacteria* tends to be greater than 1. Note that *Crocosphaera*, *Synechococcus*, and *Trichodesmium* have more histidine kinases per 100 protein-encoding genes than *Prochlorococcus*, which is adapted to highly oligotrophic conditions. The picocyanobacteria *Prochlorococcus* and *Synechococcus* have particularly high RR/HPK ratios.

We examined the TCS systems of *Proteobacteria* and *Cyanobacteria* to explore differences among phylogenetically related organisms. *Proteobacteria* tend to have many histidine kinases (Fig. 7E). As before, we found that the *Deltaproteobacteria* devote a large portion of their genome to histidine kinases (see Fig. 3). In the *Cyanobacteria*, we observed more variation, with *Prochlorococcus* and *Synechococcus* tending to have few histidine kinases and the nitrogen-fixing diazotrophs having more (Fig. 7C).

*Proteobacteria* tend to have RR/HPK ratios greater than 1 (1.03 on average), suggesting the possibility of the presence of extra response regulators in the genomes. The RR/HPK ratios of the *Cyanobacteria* are more variable than those of the *Proteobacteria*. *Prochlorococcus* and *Synechococcus*, for instance, have very high RR/HPK ratios (1.22 and 1.23, respectively), indicating that there are many extra response regulators in the genome, while *Trichodesmium* organisms have a low RR/HPK ratio (0.75), indicating the possibility of the presence of extra histidine kinases.

### Biogeochemical relevance of two-component sensory systems.

**FIG 8:**
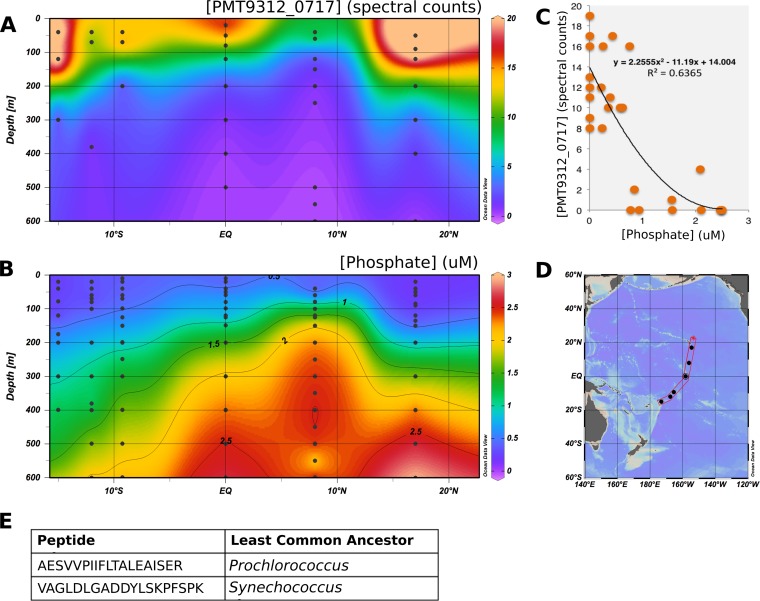
(A) Distribution of response regulator PMT9312_0717 in metaproteomes of the South Pacific Ocean. (B) Distribution of dissolved phosphate in the water column. (C) The abundance of PMT9312_0717 (measured as spectral counts) is correlated to phosphate concentrations. The relationship can be modeled by a power law. The distribution of *Prochlorococcus* cells is high across the transect (see Fig. S2). (D) Map of the METZYME transect where these samples were acquired. (E) Taxonomic information for the identified peptides. Two peptides were identified, one of which was annotated separately to two different *Prochlorococcus* strains. METATRYP analysis suggests that both of these peptides are specific to the order *Synechococcales*.

To investigate whether TCS proteins can be used as biomarkers of oceanographic processes, we examined the distribution of a putative *Prochlorococcus* MIT9312 phosphate-sensing response regulator (PMT9312_0717) in metaproteomes of the tropical Pacific (Fig. 8). Data acquisition and analysis were previously described by Saito et al. in November 2011 (16). We identified two unique peptides from this protein. Using the open-source Metatryp software package (17), we determined that one peptide is specific to *Prochlorococcus* and that the other is specific to the order *Synechococcales*. Thus, the distribution presented here can be thought of as a general picocyanobacteria signal (Fig. 8D).

10.1128/mSystems.00317-18.2 FIG S2 Hydrographic and pigment data from the METZYME cruise. The distribution of PMT9312_0717 is not well correlated with chlorophyll-a, divinyl chlorophyll-a, temperature, nor salinity in this region but is well correlated with dissolved-phosphate concentrations (see Fig. 8). Data are from M. Saito, Biological and Chemical Oceanography Data Management Office (BCO-DMO), https://www.bco-dmo.org/dataset-deployment/716688 (data set version 11 October 2017; accessed 26 October 2018), and M. Saito, Biological and Chemical Oceanography Data Management Office (BCO-DMO), https://www.bco-dmo.org/dataset-deployment/646117 (data set version 1 August 2017; accessed 26 October 2018). Download FIG S2, EPS file, 1.0 MB. Copyright © 2019 Held et al. 2019 Held et al. This content is distributed under the terms of the Creative Commons Attribution 4.0 International license.

PMT9312_0717 was confined to the upper euphotic zone and was less prevalent in phosphate-rich waters near the equator (Fig. 8A). Notably, protein abundance was inversely correlated to phosphate concentration; the relationship can be modeled with a simple power law (*r*^2^ = 0.64) (Fig. 8B and C). *Prochlorococcus* is highly abundant throughout the transect, while the protein is not, suggesting that PMT9312_0717 is specifically involved in phosphate regulation (Fig. S2). However, as with many orphan TCS systems in marine bacteria, we were unable to identify a histidine kinase with corresponding relationships to phosphate concentrations. Thus, the exact regulatory function and mechanism of PMT9312_0717 remain a mystery, despite its probable biogeochemical relevance.

## DISCUSSION

Two-component sensory systems allow bacteria to directly sense their internal and external surroundings and therefore play key roles in bacterial “intelligence” (18, 19). Because TCS systems are the most common regulatory systems in bacteria, studying them can provide insight into how individual cells might interact with their surroundings. As a result, TCS systems are of potentially significant ecological and biogeochemical importance, and yet they have been little studied in this context (7, 16). In this study, we surveyed the TCS systems of 328 diverse marine bacteria and described patterns in marine two-component sensing. We identified evidence of regulatory networking that may distinguish marine bacteria from other organisms and demonstrated that the abundance of a TCS system protein can be linked to oceanographic patterns.

### Lifestyle influences TCS gene abundance.

As in prior surveys, we found that the number of histidine kinases in the genome is largely determined by genome size (2, 4). However, the proportion of the genome encoding histidine kinases varies and is related to lifestyle and niche. The concept of copiotrophy versus oligotrophy is a common theme in microbial oceanography due to pronounced patterns in nutrient abundance versus scarcity, respectively, found in ocean environments (9–12). It is similar to K versus r selection in that oligotrophs, which are adapted to low-nutrient conditions, grow significantly slower than copiotrophs, which are adapted to high-nutrient conditions. Oligotrophs have smaller genomes and cell sizes that allow the organism to thrive in resource-limited environments (Table 1).

The small genomes of oligotrophs are thought to be the result of genome streamlining processes that are in turn driven by the need to conserve energetic and elemental resources (nitrogen and phosphorus in particular) (9, 11, 19). Our analysis suggests that the nutritional and energetic costs of maintaining TCS systems in oligotrophic environments outweigh the regulatory benefits. Lack of TCS genes has previously been observed in extremely oligotrophic organisms such as *Pelagibacter*, which may rely on simpler regulatory structures (such as one-component systems or riboswitches) which require fewer energetic and nutritional resources (20–22). The extent to which other marine oligotrophs may utilize simplified regulatory systems instead of or in addition to two-component systems is not yet known, but this survey found that the presence of few histidine kinases per gene is a hallmark of oligotrophy.

A previous effort to identify genomic signatures of oligotrophy found that histidine kinases were a weak indicator of copiotrophy versus oligotrophy (19). However, the study compared only two microbes, in contrast to the broader study across a larger number of diverse marine bacteria described here. The inability to adapt to rapid changes in nutrient availability and other environmental conditions may explain why oligotrophs are unable to survive in nutrient-rich environments (23). A recent comparison of coastal and open ocean strains of *Synechococcus* cyanobacteria supports this notion, where a coastal strain was found to have a more dynamic proteome response to iron scarcity than an oligotrophic strain (24).

In contrast to oligotrophs, certain organisms devote a comparatively large portion of their genome to histidine kinase genes. These bacteria often have specific traits such as mat formation that may require coordinated physiological alterations (25, 26). Many are sulfate or nitrate reducers from deep sea sediments or hydrothermal vents, which might represent particularly dynamic environments. Two-component systems may be particularly important for redox regulation in these organisms (27).

### Unique patterns in marine TCS systems: RR/HPK ratios, orphan genes, and hybrid systems.

We identified unique trends in the TCS system genes of marine bacteria. Specifically, we found that marine bacteria tend to have (i) higher RR/HPK ratios, (ii) many orphan TCS genes, and (iii) more hybrid histidine kinases than bacteria examined in a reference data set. We discuss each of these observations in turn.

Marine bacteria have significantly higher response regulator:histidine kinase ratios (RR:HPK) than bacteria as a whole, suggesting that they have extra response regulator genes. By considering the RR/HPK ratio, we found that approximately 1 in 50 response regulator genes lacks a histidine kinase partner (Fig. 4). The origin of the extra response regulators is puzzling. Some, lacking a histidine kinase partner, may have no regulatory function and could have been the result of incomplete genetic innovations or horizontal gene transfers (HGT). However, this explanation is not consistent with the tendency toward genome streamlining in the nutrient-limited ocean environment (9, 11). An intriguing alternative is that some of the extra response regulators might participate in regulatory networks in which multiple response regulators interact with a single histidine kinase. Such networks are a topic of increasing study and have been implicated in coordinating nutrient acquisition (specifically, acquisition of phosphate and iron), sporulation processes, stress response, and circadian rhythms (28–31).

Colocalization of TCS genes is thought to provide for concerted transcription of the sensory genes and better success in HGT (15, 32). Marine bacteria seem to be an exception to this rule, having many orphan genes in their genomes (Fig. 5). Orphan TCS genes are present in even the most streamlined genomes (i.e., that of *Pelagibacter ubique*), suggesting that they play important biochemical roles. The nonmodularity of TCS systems in marine bacteria suggests that the genes are not acquired through HGT but are instead acquired through gene duplication and genetic remodeling (32). TCS systems created in this way are thought to be more likely to participate in regulatory cross talk than systems that are acquired through horizontal gene transfer (3). Indeed, orphan genes are often involved in essential regulatory networks in model organisms (33–36). Alternatively, it is possible that in situations in which regulatory networks occur, the relationship between HPK and RR is not as specific as in normal two-component systems. This lack of specificity could allow nonmodular TCS genes to become fixed in the genome. Most of what we know about two-component systems is based on studies of modular systems from model organisms; additional studies on non-model organisms may thus reveal new mechanisms of acquisition and action of TCS genes.

In hybrid histidine kinases, the phosphorylation signal is relayed by one or more histidine phosphotransfer (Hpt) proteins before it reaches a terminal response regulator (Fig. 1). The added complexity of the phosphorelay is thought to provide multiple points of regulation, allowing fine-tuned physiological responses. For example, a hybrid histidine kinase regulates glycan utilization in Bacteroides thetaiotaomicron by integrating both intracellular metabolism and extracellular substrate signals (37). Hybrid histidine kinases are associated with physiological and behavioral complexity, being especially prevalent in higher eukaryotes (38). Marine bacteria such as *Proteobacteria* and *Bacteroidetes* can have many hybrid TCS systems (Fig. 5; see also Table 1), concurrent with a tendency toward metabolic complexity and particle association, which may drive multicellular behaviors (28).

Together, the presence of extra response regulators, the nonmodularity of TCS systems, and the prevalence of hybrid TCS systems suggest increased regulatory complexity in marine bacteria. This may confer advantages in the ocean environment, where bacteria are often chronically nutrient limited. In the ocean, nutrient availability is determined by diffusion rates, resulting in covariance in the distribution of organic nitrogen, carbon, phosphorus, and trace nutrients, especially at the microscale (28, 39). Nutrient colimitation has been demonstrated in a number of marine environments and may be more prevalent than originally thought (40–42). Marine organisms appear to have specific physiological responses to colimitation, for example, proteome restructuring and cell size decreases in iron and phosphate colimited *Trichodesmium* cells (43). Although the regulatory systems for colimitation in marine microbes have yet to be elucidated, a precedent for regulatory networking has been identified in nonmarine organisms such as Edwardsiella tarda, in which the Pho and Fur systems interact with one another (31). The irregularities in marine bacterial TCS genes suggest that similar regulatory networks may underpin concerted responses to multiple environmental perturbations.

### Comparison of *Proteobacteria* and *Cyanobacteria*.

*Proteobacteria* and *Cyanobacteria* are perhaps the most abundant and well-studied cells in the ocean. Comparing them demonstrates the impact of lifestyle traits on the number of histidine kinases in the genome. With the exception of the oligotrophic *Pelagibacter* species, the *Proteobacteria* have a larger proportion of genes encoding histidine kinases (0.95 per 100 protein-encoding genes on average) than the *Cyanobacteria* (0.57 per 100 protein-encoding genes on average) (Fig. 7A and C). This may be related to their tendency toward copiotrophy, which is associated with large numbers of TCS system genes. Within the *Cyanobacteria*, nitrogen-fixing organisms have more histidine kinases. Diazotrophs are not subjected to the same genome streamlining pressure as other marine *Cyanobacteria* owing to the fact that they can access an unlimited supply of atmospheric nitrogen. However, the complexities of the diazotrophic lifestyle may necessitate a large number of regulatory genes. For instance, nitrogen fixation rates are known to respond to many environmental parameters such as iron nitrogen, phosphorus, dust, and light availability (44–48). Reflecting this, marine diazotrophs have two-component systems regulating nutrient availability, complex circadian rhythms, and redox state.

Evidence for regulatory networking is prevalent in both the *Proteobacteria* and *Cyanobacteria* (Fig. 7B and D). Most *Proteobacteria* and picocyanobacteria have elevated RR/HPK ratios that suggest the presence of branched regulatory networks in which one histidine kinase communicates with multiple response regulators. Branched regulatory networks could allow multiple operons to be affected by a single sensory input, with each chemical sensor triggering multiple downstream effects, providing greater metabolic flexibility and dynamism. This could provide for fine-tuned responses to multiple stimuli, such as nutrient colimitation. In nutrient-limited environments, regulatory networking may provide an advantage for cellular resource conservation. For instance, a single stimulus can trigger multiple physiological reactions without the need to express an entire two-component system for each operon. Consistently, we found that the oligotrophic genomes from organisms such as *Pelagibacter* and *Prochlorococcus* have especially high RR/HPK ratios (Table 1).

Notably, the diazotrophic organism *Trichodesmium* has low RR/HPK ratios, suggesting regulatory networking in which multiple histidine kinases interact with a single response regulator. This may allow integration of multiple environmental signals in regulating a single physiological response and may underpin extensive proteomic changes such as coordination of carbon and nitrogen fixation processes over the course of the diel cycle (49). However, identifying these networks is challenging because the majority of two-component-system genes in *Trichodesmium* (and many marine bacteria) have not been characterized.

### Two-component systems as potential biogeochemical biomarkers.

Having so far considered TCS genes, we next turned to the gene products and their relationship to oceanographic processes. We studied a *Prochlorococcus/Synechococcus* PhoB-like response regulator protein, PMT9312_0717, in the tropical Pacific Ocean. PMT9312_0717 is one of the most abundant TCS system proteins in this transect. It is an orphan, and its partner histidine kinase is not known. In the metaproteomics analysis, we found that the protein abundance is inversely correlated with inorganic phosphate concentration, particularly at concentrations below 1 μM phosphate such as are encountered near the nutricline (Fig. 8). The abundance of *Prochlorococcus* cells is high throughout the transect, while that of PMT9312_0717 is not and instead follows trends in phosphate concentrations (see Fig. S2 in the supplemental material). This implies that the abundance of the protein is related to oceanographic processes, suggesting that the protein self-regulates its production. Increased abundance of TCS systems in response to low dissolved phosphorus concentrations has been previously observed for phosphate-regulating systems (7, 16). Measurement of levels of protein phosphorylation (i.e., the activity of the TCS system), while technically difficult, could provide additional information about the function of the protein.

In addition to this protein distribution, genomic evidence also suggests that PMT9312_0717 is involved in phosphate regulation. PMT9312_0717 is present in many *Prochlorococcus* species, including both high- and low-light ecotypes, as well as in *Synechococcus* species. It is similar to both the *Prochlorococcus* sp. 9312 phosphate-sensing histidine kinase PhoB (37% identity) and the analogous *Synechococcus* sp. WH8102 PhoP protein (WP_025362545.1; 37% identity) (16) but is a distinct protein. Because just a few point mutations can change the function of a response regulator, we hypothesized that this protein participates in phosphate regulation but in ways that differ from those seen with PhoB/PhoP. Corroborating its possible role in phosphate sensing, PMT9312_0717 is located near a DedA family alkaline phosphatase-related gene (e.g., PMT9312_0712) in multiple strains of *Prochlorococcus*. TCS systems have amino acids known as specificity residues that govern the histidine kinase-response regulator interaction (4). The specificity residues of the PMT9312_0717 and PhoB genes in *Prochlorococcus* sp. MIT 9312 are not shared, indicating that the PMT9312_0717 is unlikely to interact with the phosphate-sensing PhoR histidine kinase.

TCS systems underpin many of the physiological changes observed in laboratory and field perturbations of marine bacteria. They are involved in nutrient acquisition, detoxification, quorum sensing, and other topical themes in marine microbiology. However, our knowledge of these systems in marine species is limited. Sequence-based identification of TCS systems is difficult because histidine kinases and response regulators share highly conserved catalytic domains. Thus, while it is possible to identify TCS genes, identifying their physiological functions is tricky. Few two-component systems have been experimentally verified in marine species, despite the fact that just a few amino acid substitutions can drastically change physiological function (4).

The environmental drivers behind gain/loss of two-component sensory system genes and protein synthesis processes are not well understood. For instance, distribution of the phosphate-sensing *phoR*-*phoB* two-component genes in *Prochlorococcus*, while initially hypothesized to be correlated to phosphate availability, cannot be consistently linked to large-scale oceanographic patterns (7, 50). An important consideration is that two-component sensory systems act on short time scales—seconds, minutes, or hours (51, 52). The presence of a TCS system thus suggests the need to continuously monitor the stimulus. For nutrient-sensing regulators, it may be more accurate to suggest that distribution of the TCS system is related to varying (not necessarily chronically depleted) nutrient concentrations such as are found in surface waters. This is corroborated by our finding that the amount of PMT9312_0717 protein in the water column increases significantly near nutricline like concentrations. Given this circumstantial and yet consistent evidence, a detailed biochemical characterization of PMT9312_0717 is an intriguing topic for future study. However, on the basis of this and previous work, it is clear that TCS system protein abundances can contain valuable biogeochemical information (15).

### Conclusions.

Two-component sensory systems reveal characteristics of both individual cells and the ecosystems in which they live. In this way, they represent a unique opportunity to link microbial physiology to the environment. We know relatively little about TCS systems in marine bacteria, but it is clear that the distribution of TCS genes and proteins is dependent on both the traits of the organism and its surrounding environment. For instance, we found that oligotrophs have significantly fewer histidine kinases per gene than copiotrophs and that diazotrophy is associated with greater numbers of TCS system genes. Importantly, we found that marine microbes may have adapted unique ways to sense their environments using complex regulatory networks. Additional characterization of these networks may provide us with a greater appreciation for both the uniqueness of the ocean environment and the breadth of sensory systems used by prokaryotes. Detailed biochemical characterization of marine two-component systems is greatly needed and has great potential to advance our understanding of microbial life and its connections to global biogeochemical cycles.

## MATERIALS AND METHODS

### Marine and reference bacteria data sets.

We acquired the genomes of 328 marine bacteria available in the JGI IMG data warehouse (see Table S1 in the supplemental material). All of the genomes were high-quality finished genomes or permanent drafts. We note that permanent draft genomes could be missing some RR or HPK genes due to incomplete assembly, but this is not expected to significantly affect results. The marine data set is phylogenetically diverse; the best represented phyla are the *Cyanobacteria*, *Proteobacteria*, *Firmicutes*, *Bacteroidetes*, and *Actinobacteria* (see Fig. S1 in the supplemental material). The bacteria were isolated from various habitats that included coastal ecosystems, the open ocean, hydrothermal vent systems, marine sediments, and microbial mats. Most of the genomes that we examined were from bacterial isolates in culture; as such, bacteria of particular oceanographic interest such as *Prochlorococcus*, as well as easily cultivated organisms such as *Alteromonas*, form a large fraction of the data set. While we attempted to capture maximal phylogenetic and habitat level variability, this data set does not necessarily represent the actual levels of bacterial diversity nor cell abundance in the ocean environment. Rather, the intention was to allow us to examine the marine bacteria for which we have good-quality genomic information at this time.

The reference bacteria data set was largely derived from the organisms associated with the GEBA-I initiative, which provides a collection of bacterial genomes spanning the breadth of known phylogenetic diversity (Table S2) (8). The GEBA-I database includes genomes of both bacteria and archaea; we used only the bacterial genomes here. We observed that *Cyanobacteria* are underrepresented in the GEBA-I data set relative to the marine data set described above. To facilitate comparisons between the marine and reference data sets, we included 180 high-quality, nonmarine *Cyanobacteria* genomes from the JGI IMG warehouse in the reference data set.

### Identification of two-component-system genes.

We identified TCS system genes by protein family (pfam) domain annotations (53). The pfam domain annotation was performed for each genome as part of their initial ingestion into the IMG warehouse. Comparisons were thus facilitated by the fact that all genomes were subjected to similar annotation analysis pipelines in the JGI IMG portal.

Histidine kinases are composed of two domains—an HATPase domain and a phosphoacceptor domain—which represent separate entries in the pfam domain database. We tested both the HATPase and phosphoacceptor domains by comparing the number of histidine kinases identified with the results of an extensively curated previously published survey of TCS genes in bacteria (18). We found that the HATPase domain was better conserved, identified more histidine kinases, and resulted in histidine kinase identifications comparable to those in the previous study. Thus, we used the HATPase domain for histidine kinase identification subsequently. The specific pfams used were pfam02518 (HATPase_c), pfam13581 (HATPase_c_2), pfam13589 (HATPase_c_3), pfam14501 (HATPase_c_5), and pfam07536 (HWE_HK). The HATPase domain is also present in proteins such as DNA gyrase (pfam00204), HSP90 (pfam00183), and MutL (pfam13941); we removed genes containing these three pfams from the analysis.

Response regulators are composed of a phosphoreceiver domain (sometimes known as a REC domain) and an output domain. The output domain can vary significantly and determines the biological function of the response regulator (52, 54). We used the protein family domain for the response regulator receiver (pfam00072) to identify response regulators in our data sets. When possible, we compared the results of this pfam-based identification of response regulators to that of a previously published survey and found that the results of the pfam-based analysis were comparable (18).

Hybrid histidine kinases occur when the response regulator phosphoacceptor domain is present on the same protein as the histidine kinase sensory and phosphoacceptor domains. We define them here as genes containing both a HATPase domain (pfam02518, pfam13581, pfam13589, pfam14501, or pfam07730) and the response regulator phosphoreceiver domain (pfam00072). Thus, the hybrid histidine kinases are present in both the histidine kinase data and response regulator data for a given genome.

### Statistical and meta-analyses.

All analyses were conducted in Python 3.6. The entire data analysis and visualization pipeline, including statistical analyses and machine learning algorithms, can be recreated by accessing the scripts at https://github.com/naheld/patterns_TCS_sensing_marine_bacteria. A fully executable cloud environment is provided courtesy of the Binder project (https://mybinder.org/). We used the Bokeh (https://bokeh.pydata.org/en/latest/) and seaborn (https://seaborn.pydata.org/) libraries to generate visualizations and perform statistical analyses. For ratio and statistical analyses, genomes containing 0 histidine kinases and/or 0 response regulators were excluded.

For K means clustering analyses, we counted the number of histidine kinases, including hybrid genes, and normalized the data by dividing by the number of protein-encoding genes. For human readability, we express this value as the number of histidine kinases per 100 genes. To eliminate the effect of scaling, we subjected the data to unit normalization. We then performed the clustering analysis to identify groups of genomes with similar signaling repertoires in relation to genome size. We used the Silhouette method implemented in the Scikit Learn Cluster module to select the optimal number of clusters (4) by maximizing the average Silhouette coefficient values (55). Clustering analysis was performed with the SciKit Learn K-means clustering algorithm.

### Locations of TCS genes.

The locations of TCS genes in the genomes of marine bacteria were examined by plotting the gene starting locations on a linearized depiction of the circular bacterial genome (a number line). The average gene size was calculated for each genome examined. A gene was considered to be an orphan if its start point was farther than four average gene lengths from another TCS gene.

### PhoB protein distribution analysis.

We examined the distribution of response regulator PMT9312_0717 in marine metaproteomes from the METZYME expedition in the tropical Pacific. Analysis of these metaproteomes has been previously described (16). Briefly, microbial biomass was collected with *in situ* particle collection pumps (McLane Labs) on the KM1128 METZYME research expedition. Proteins from the 0.2-uM to 3-uM size fraction were subjected to SDS detergent extraction and in-gel trypsin digestion as previously described (16). Peptides were analyzed by liquid chromatography-tandem mass spectrometry (LC-MS/MS) using 1-dimensional chromatography on a Thermo Fusion Orbitrap mass spectrometer (16). Spectral counts for the protein were generated by mapping against 6 metagenomes sampled from the METZYME expedition. These were sequenced at JGI and assembled using metaSPAdes (56). Genes were predicted and annotated using the pipeline described previously by Dupont et al. (57). Peptide-to-spectrum matches (PSMs) were identified by SEQUEST and restricted to a 10-ppm peptide mass threshold and a 99.0% protein probability threshold (16). Two unique peptides were identified for PMT9312_0717; we performed redundancy analysis using the openly available Metatryp software (17). Corresponding inorganic phosphate analyses were conducted by Joe Jennings at Oregon State University as previously described (58).
